# The emergence of *RAS* mutations in patients with *RAS* wild-type mCRC receiving cetuximab as first-line treatment: a noninterventional, uncontrolled multicenter study

**DOI:** 10.1038/s41416-023-02366-z

**Published:** 2023-07-24

**Authors:** Hsiang-Lin Tsai, Chun-Chi Lin, Yung-Chung Sung, Shang-Hung Chen, Li-Tzong Chen, Jeng-Kai Jiang, Jaw-Yuan Wang

**Affiliations:** 1 Division of Colorectal Surgery, Department of Surgery, Kaohsiung Medical University Hospital, Kaohsiung Medical University, Kaohsiung, Taiwan; 2 Department of Surgery, Faculty of Medicine, College of Medicine, Kaohsiung Medical University, Kaohsiung, Taiwan; 3 Division of Colorectal Surgery, Department of Surgery, Taipei Veterans General Hospital, Taipei, Taiwan; 4 Department of Surgery, School of Medicine, National Yang Ming Chiao Tung University, Taipei, Taiwan; 5 School of Medicine, Fu-Jen Catholic University, New Taipei City, Taiwan; 6 Division of Hematology/Oncology, Internal Medicine, Cathay General Hospital, Taipei, Taiwan; 7 Division of Hematology and Oncology, Department of Internal Medicine, National Cheng Kung University Hospital, College of Medicine, National Cheng Kung University, Tainan, Taiwan; 8 National Institute of Cancer Research, National Health Research Institutes, Tainan, Taiwan; 9 Division of Medical Oncology, Department of Internal Medicine, Kaohsiung Medical University Hospital, Kaohsiung Medical University, Kaohsiung, Taiwan; 10 Department of Internal Medicine, Faculty of Medicine, College of Medicine, Kaohsiung Medical University, Kaohsiung, Taiwan; 11 Graduate Institute of Medicine, College of Medicine, Kaohsiung Medical University, Kaohsiung, Taiwan; 12 Center for Cancer Research, Kaohsiung Medical University, Kaohsiung, Taiwan; 13 Pingtung Hospital, Ministry of Health and Welfare, Pingtung, Taiwan

**Keywords:** Cancer therapeutic resistance, Molecular biology

## Abstract

**Background:**

Patients treated with anti-epidermal growth factor receptor (anti-EGFR) will ultimately develop acquired resistance promoted by clonal selection, mainly the emergence of mutations in the MAPK pathway (mostly *RAS* mutations). Baseline assessment of *RAS* mutations in the blood of patients correlates well with *RAS* tumour tissue testing and is currently an alternative option in routine clinical practice to guide first-line therapy. The aim of this study was the prevalence of acquired genomic alterations detected in the auxiliary tool of ctDNA testing and investigated the role of *RAS* ctDNA status for detecting tumour response and predicting benefit to anti-EGFR therapy.

**Methods:**

Only patients with concordant wild-type formalin-fixed, paraffin-embedded (FFPE) tumour tissue and baseline ctDNA *RAS* wild-type were included. *RAS* mutations in plasma were evaluated using MassARRAY platform. Blood samples were collected at baseline, every 3 months during first-line treatment, and at disease progression. The primary endpoint was the detection rate of *RAS* mutations during cetuximab treatment. The correlation between response and survival outcomes and the emergence of circulating *RAS* mutations was also analysed.

**Results:**

The detection rate of *RAS* mutations during treatment was 9.3% (10/108). *RAS* mutations detection occurred a median of 3 months prior to radiologic documentation. The subgroup of patients with *RAS* mutations exhibited significantly inferior progression-free survival and overall survival (*P* = 0.002 and 0.027, respectively) but the baseline characteristics, response rates, disease control rates, and metastatectomy were not significant (all *P* > 0.05).

**Conclusions:**

We demonstrated that *RAS* ctDNA status might be a valuable biomarker for detecting early tumour response and predicting benefit to anti-EGFR therapy.

Clinical Trial Registration: NCT03401957 (January 17, 2018).

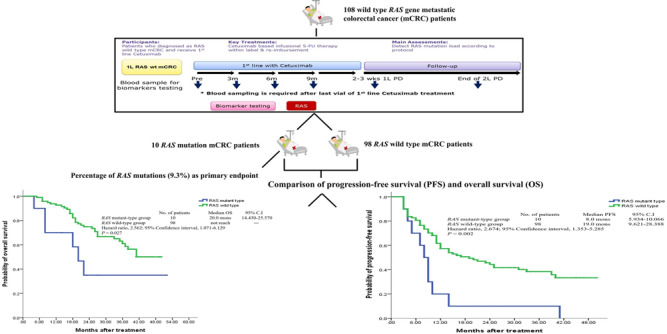

## Introduction

Colorectal cancer (CRC) is the third most prevalent form of cancer and the second largest cause of cancer-related deaths globally [1]. In total, 25% of patients with new diagnoses of CRC have metastatic CRC (mCRC), and 25–30% of patients with new diagnoses of stage I–III CRC eventually develop mCRC [2–5]. In addition to conventional chemotherapeutic drugs, several agents targeting the molecular drivers of CRC pathogenesis, including signalling pathways mediated by epidermal growth factor receptor (EGFR) and vascular endothelial growth factor (VEGF), have been widely administered to patients with mCRC, with the result being increasing survival rates [6–8].

EGFR is a key factor in cellular proliferation, differentiation, and survival [9]; thus EGFR-targeted therapy is used in malignancy treatment [10]. The use of cetuximab and panitumumab, two monoclonal antibodies (mAbs) directly targeting EGFR, can prolong the survival of patients with mCRC [11] and enable metastatectomy [12]. Although treatment with anti-EGFR agents and chemotherapy exert considerable effects against mCRC, drug resistance limits clinical application—as treatment progresses, approximately 80% of responders subsequently develop drug resistance [13]. The mechanisms of resistance to anti-EGFR agents include gene mutations downstream of the EGFR signalling pathway, such as mutations of *RAS/RAF/MEK* and *PIK3/AKT/mTOR*, which contribute significantly to drug resistance [14, 15].

Per clinical guidelines, molecular assays used for clinical decision-making are based on tumour biopsies, which represent the gold standard [16, 17]. However, several limitations of single solid tissue biopsies have been reported, such as spatial and temporal tumour heterogeneity and technical feasibility issues [18–20]. Burrell et al. also reported that the development of drug resistance within tumour cells is believed to be a dynamic process of ecological evolution [21]. A liquid biopsy is the collection of small tumour-derived pieces of DNA or RNA or other molecules in the bloodstream, urine, saliva, stool, or cerebrospinal fluid [22–24]. The most common assessments of tumour-related biomarkers in liquid biopsies include those of circulating tumour DNA (ctDNA), circulating tumour cells (CTCs), and exosomes [22, 25]. Among these, ctDNA analysis, consisting of the isolation of DNA fragments from the bloodstreams of patients, exhibits potential, as it can capture CRC molecular complexity, and the technical advantages of minimal invasiveness and rapid turnaround [22, 26]. Liquid biopsy analysis of ctDNA avoids the limitations of tumour tissue–based mutation analysis. Although this minimally invasive technique offers the advantage of continual monitoring of the major genotype present in tumour cells with complex heterogeneity, there were some limitations to prevent the use of ctDNA as a clinical tool in the routine care of patients with mCRC at current time. The limitations of ctDNA include (1) sophisticated downstream analysis of ctDNA requires expert skills; (2) when the tumour burden is very low, ctDNA molecules can also become a limiting factor in early cancer detection; (3) decreases in ctDNA level during systemic therapy (first or second line of therapy) correlate with tumour response in the CRC metastatic setting; (4) before entering clinical practice, it is necessary to prove the clinical utility of ctDNA, and this can only be achieved in international clinical trials where the biomarker results determine the treatment choice [27, 28]. The clinical use of ctDNA as a biomarker in cancer care will depend on the standardisation of pre-analytic and analytic procedures [27].

A mass spectrometry–based technique combined with single-base extension polymerase chain reaction (PCR) was used to investigate genotyping in a variety of human cancers [29]. The mass spectrometry platform used for this high-throughput technique, the MassARRAY platform (Sequenom, Brisbane, Australia), has been used to verify the concordance of genotyping in patient-matched plasma and tumour tissue samples from patients with CRC [30, 31]. In this prospective multicenter study, we used this platform to identify *RAS* mutations in serial blood samples collected from patients with mCRC receiving cetuximab-based therapy as first-line treatment. The aim of this study focused to investigate the prevalence of acquired genomic alterations and the role of *RAS* ctDNA status for detecting early tumour response and predicting benefit to anti-EGFR therapy.

## Methods

### Study design

This investigator-initiated trial (IIT) was a single-arm, noninterventional, uncontrolled multicenter study performed in four member hospitals of the Colorectal Cancer Consortium in Taiwan. The definition of mCRC was metachronous or synchronous adenocarcinoma with distant metastasis. Patients with *RAS* wild-type mCRC diagnoses after formalin-fixed, paraffin-embedded (FFPE) tumour tissue and ctDNA examination were recruited, and the emergence of *RAS* mutations in patients with mCRC receiving a cetuximab-based regimen as first-line treatment was evaluated. In addition to cetuximab, infusional 5-fluorouracil (5-FU) in combination with oxaliplatin or irinotecan as first-line treatment was required for inclusion. Treatment was continued until disease progression, the occurrence of intolerable toxic effects, or withdrawal of consent. Blood samples were collected before the start of treatment and every 3 months during first-line treatment. When disease progression occurred, blood sampling was also required within 3 weeks following cetuximab and second-line treatments. The blood samples were sent to the central laboratory at Taipei Institute of Pathology and tested for the *RAS* genotype using the MassARRAY platform combined with the single allele base extension reaction (SABER) technique (Agena, San Diego, California, USA). Pretreatment tissue sections were re-evaluated for the *RAS* genotype using this technique if inconsistency regarding *RAS* was detected between the tissue and blood samples of the same patient. The study design, patient characteristics, inclusion and exclusion criteria, and detailed treatment regimens, including patient withdrawal data, were described in our study protocol [32]. This protocol is briefly outlined in Supplementary Fig. 1.

Written informed consent was obtained from each participant. The trial was conducted in accordance with the Guideline for Good Clinical Practice and the Declaration of Helsinki; approved by the Institutional Review Board of Kaohsiung Medical University Hospital [KMUHIRB-G(II)-20170027], Taipei Veterans General Hospital (reference number: 2017–12-003A), Cathay General Hospital (reference number: CGH-P107013), and National Cheng Kung University Hospital (reference number: A-BR-106-045); and registered at ClinicalTrials.gov (NCT03401957).

### Enroled patient numbers

Studies have demonstrated that 21–33% of patients with *RAS* wild-type mCRC at baseline exhibited *KRAS* mutations at weeks 24 and 26, respectively [33, 34]. With consideration for test power, we used a confidence limit to calculate the sample size. Using the Wilson score method, a sample size of 110 produced two-sided 95% confidence intervals (CIs) of 0.169, 0.176, and 0.180 with sample proportions of 0.300, 0.350, and 0.400, respectively [35]. Accounting for a 10% dropout rate, enrolment of approximately 120 patients was considered appropriate.

### Blood sampling

In total, 20 mL of blood was obtained from an arterial or venous line using a standard phlebotomy technique, with two 10 mL cell-free DNA (cfDNA) collection tubes (Roche Diagnostics Ltd.) used for sampling. Blood specimens were shipped at room temperature within 24 h. Plasma and ctDNA were extracted using a ctDNA sample preparation kit (cobas) and were processed within 7 days after blood drawing at the Taipei Institute of Pathology.

### Schedule of blood sampling assessment

The enroled patients received *RAS* mutation analysis of ctDNA every 3 months during cetuximab-based first-line treatment and within 3 weeks of disease progression after first-line treatment. During the study period, patient assessment was scheduled according to the clinical judgement of the responsible investigator.

### MassARRAY technique

All DNA samples extracted from blood or tissue were frozen at –20 °C before mutation analysis. ctDNA was extracted using a ctDNA sample preparation kit (cobas). Three to five sections of formalin-fixed paraffin-embedded (FFPE) tissue were used for DNA extraction with a DNA FFPE tissue kit (QIAamp). Mutation detection was performed using the SABER method on a MassARRAY platform (Agena). The SABER reaction used the iPLEX enzyme, SABER terminator mix, and an extension primer mix (iPLEX Pro Kit, Agena). The SABER method restricts primer extension to the allele of interest and improves detection sensitivity. The SABER reaction was intentionally undertaken to not include terminators for the WT nucleotide, only mutant terminators.

Multiplex PCR was used to amplify the targeted region. In total, 1 μL of cfDNA was loaded in 384-well PCR plates. The reaction mixture included dNTPs, primer pools (forward and reverse), reaction buffers, and DNA polymerase. After the first PCR, the reaction mixture was treated with shrimp alkaline phosphatase, and single-base extension was then performed using the iPLEX enzyme, SABER terminator mix, and extension primers (pooled single extension primers). PCR conditions were applied in accordance with Agena Bioscience iPLEX SABER guidelines.

After the final PCR, the product (7 nL reaction mix) underwent resin treatment and was transferred to a 384-well Spectro CHIP array using the system nanodispenser. The product was then subjected to mass spectrometry analysis (MassARRAY Analyzer 4), and the data obtained were analysed using the preinstalled Typer Analyzer 4.0 software (Agena). One positive and one negative control were added to the 384-well plate for quality control. The mutation base extension ratio was calculated as the mutation extension peak area percentage of the unextended and extended peak area. The baseline for each mutation assay was established using a wild-type sample pool. The cutoff value was set as a standard deviation (SD) of five above the baseline value.

In this study, all participants received routine *KRAS* (codon 12, 13, 59, 61, 117, and 146) and *NRAS* (codon 12, 13, 59, 61, 117, and 146) test by MassARRAY technique.

#### Genotyping quality control

Detection of all 11 mutations of the positive control was necessary. Detection of no mutations in the negative control in the batch experiment was also required.

### Efficacy measures

Tumour responses were typically assessed after every six cycles of the interventional regimen. Response measurements are detailed in our protocol [32], and were based on the Response Evaluation Criteria in Solid Tumours (RECIST) version 1.1 [36].

### Study endpoints

The primary endpoint was the percentage of *RAS* mutations detected in the ctDNA of patients with mCRC during first-line cetuximab exposure. The secondary endpoints were as follows: (1) the time to onset of newly detected *RAS* mutant ctDNA; (2) the median interval between *RAS* mutation and disease progression; (3) the percentage of detected *RAS* mutant ctDNA at the time of progression; (4) clinical objective response rates; (5) metastasis resection rates of patients with *RAS* mutations; (6) the duration of cetuximab treatment of patients with *RAS* mutations; (7) the median progression-free survival (PFS) of the per-protocol population; and (8) the overall survival (OS) of the per-protocol population. PFS was defined as the time from the date of enrolment until the first documentation of disease progression, regardless of the patient’s treatment status. OS was defined as the time from the date of enrolment until the date of death or the last date of follow-up.

### Statistical analysis

All patients receiving at least 12 weeks or six cycles of treatment and having at least one postbaseline *RAS* mutation in their ctDNA were eligible for clinical efficacy and outcome evaluation. Continuous variables are presented as the mean ± SD, and dichotomous variables are presented as numbers and percentages. All statistical analyses were performed using SPSS Statistics version 21.0 (SPSS, IBM, Armonk, NY, USA). The clinicopathological characteristics of the two groups were compared using Pearson’s chi-squared test. Logistic regression was used to estimate the odds ratios for all independent variables in the model. PFS and OS were evaluated using the Kaplan–Meier method, and the log-rank test was used to compare time-to-event distributions.

## Results

### Patients

**Fig. 1:**
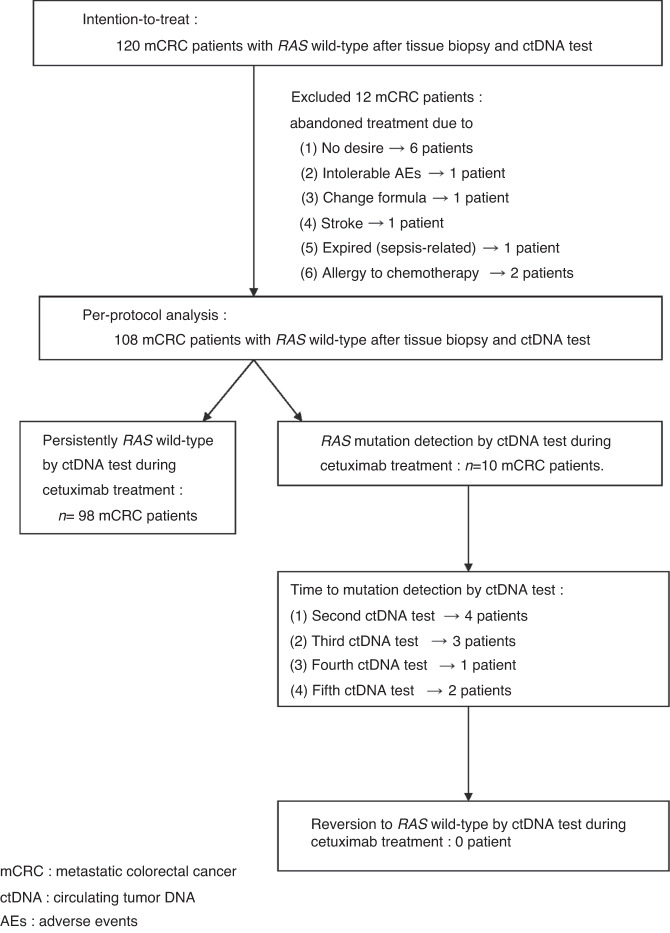
CONSORT diagram of clinical trial. Intention-to-treat population was 120 patients with metastatic colorectal cancer (mCRC), and per-protocol analysis involved 108 patients. Collection was from January 2018 to June 2020, and database was locked for final analysis on June 30, 2020. Median follow-up time was 26.5 months (interquartile range [IQR], 17.0–37.0 months). Reversion to *RAS* wild-type by ctDNA testing was 0% during the cetuximab-based treatment.

In January 2018, the first eligible patient with mCRC was screened, and in June 2020, the 120th potentially eligible participant was enroled. According to the protocol, 120 patients with mCRC were enroled for intention-to-treat (ITT) analysis, and 108 patients received per-protocol analysis. A CONSORT diagram of the study is shown in Fig. 1. There were 12 patients with mCRC to be excluded for individual reasons. Based on the integrity of statistical data, per-protocol analysis was performed.

The database for the final analysis was locked on June 30, 2022. At the cutoff time for analysis, the median follow-up was 26.5 months (interquartile range [IQR], 17.0–37.0 months). Twelve patients with mCRC were excluded because (1) six patients had no desire to continue treatment after enrolment; (2) one patient experienced intolerable adverse effects (AEs); (3) one patient changed his chemotherapy form to capecitabine; (4) one patient had a stroke episode during the treatment period; (5) one patient expired from sepsis; and (6) two patients were allergic to the chemotherapy treatment (Fig. 1). The baseline characteristics of the ITT population (120 patients with mCRC) and the per-protocol analysis population (108 patients with mCRC) are shown in Table 1. The median age was 65.0 years (range, 24.0–88.0 years) and the baseline characteristics were similar in both populations (Table 1).

**Table 1:** Baseline characteristics of the intention-to-treat population (120 patients) and of the per-protocol analysis population (108 patients).

	Intention-to treat	Per-protocol analysis
	(*n* = 120)	(*n* = 108)
Age (y/o)^a^	65.0 (24.0–88.0)	65.0 (24.0–88.0)
Gender		
Male	89 (74.2%)	80 (74.1%)
Female	31 (25.8%)	28 (25.9%)
ECOG^b^ status		
0	26 (21.7%)	24 (22.2%)
1	93 (77.5%)	83 (76.9%)
2	1 (0.8%)	1 (0.9%)
Type of mCRC^c^		
Synchronous	72 (60.0%)	62 (57.4%)
Metachronous	48 (40.0%)	46 (42.6%)
Primary tumour sidedness		
Right-sided^d^	10 (8.3%)	8 (7.4%)
Left-sided^e^	110 (91.7%)	100 (92.6%)
Regimen of chemotherapy		
FOLFIRI^f^	105 (87.5%)	95 (88.0%)
FOLFOX^g^	15 (12.5%)	13 (12.0%)
Metastatic sites		
Liver	63 (52.5%)	57 (52.8%)
Lungs	18 (15.0%)	16 (14.8%)
Peritoneum	5 (4.2%)	3 (2.8%)
Pelvis	3 (2.5%)	3 (2.8%)
Bone	1 (0.8%)	1 (0.9%)
Ovary	2 (1.7%)	2 (1.9%)
Adrenal gland	1 (0.8%)	1 (0.9%)
Para-aortic LNs	8 (6.7%)	8 (7.4%)
Common iliac LNs	1 (0.8%)	1 (0.9%)
Liver + lungs	12 (10.0%)	11 (10.2%)
Liver + bone	2 (1.7%)	1 (0.9%)
Liver + peritoneum	2 (1.7%)	2 (1.9%)
lungs + ovary	1 (0.8%)	1 (0.9%)
lungs + peritoneum	1 (0.8%)	1 (0.9%)
No.^h^ of metastases		
One	102 (85.0%)	92 (85.2%)
≧2	18 (15.0%)	16 (14.8%)
*BRAF* genotyping		
Wild type	117 (97.5%)	106 (98.1%)
Mutant type	3 (2.5%)	2 (1.9%)

^a^*y/o* year-old.

^b^*ECOG* Eastern Cooperative Oncology Group.

^c^*mCRC* metastatic colorectal cancer.

^d^*Right-sided* including cecum + ascending colon + transverse colon.

^e^*Left-sided* including descending colon + sigmoid colon + rectosigmoid junction + rectum.

^f^*FOLFIRI* Fluorouracil + leucovorin + irinotecan.

^g^*FOLFOX* Fluorouracil + leucovorin + oxaliplatin.

^h^*No.* Number.

### Emergence of *RAS* gene mutations among 108 per-protocol patients with mCRC during treatment and follow-up

Analysis of the 108 per-protocol patients until June 30, 2022, is shown in Table 2. *RAS* mutations occurred in 10 patients (9.3%) during cetuximab-based treatment. The detailed baseline characteristics of the 10 patients with *RAS* mutations during cetuximab-based first-line treatment are listed in Supplementary Table 1. The median age was 68.0 years (range, 51.0–76.0 years). Of the patients, 80% were men, and all had left-sided mCRC (100%). The most common mutation point was *G12D* of the acquired *KRAS* gene (40.0%).

**Table 2:** Clinical outcomes of per-protocol population during the period of treatment and follow-up.

	Per-protocol population
	(*n* = 108)
Best response	
Complete response (CR)	0 (0%)
Partial response (PR)	64 (59.3%)
Stable disease (SD)	24 (22.2%)
Progressive disease (PD)	20 (18.5%)
Objective response rate (ORR)	
CR + PR	64 (59.3%)
SD + PD	44 (40.7%)
Disease-control rate (DCR)	
CR + PR + SD	88 (81.5%)
PD	20 (18.5%)
Metastatectomy	
Yes	17 (15.7%)
No	91 (84.3%)
Progression during 1st-line treatment	
Yes	48 (44.4%)
No	60 (55.6%)
Progression after 1st-line treatment	
Yes	68 (63.0%)
No	40 (37.0%)
*RAS* mutation during cetuximab treatment	
Yes	10 (9.3%)
No	98 (90.7%)

The clinical outcomes of the 10 patients with *RAS* mutations are presented in Table 3. The median duration of cetuximab-based first-line treatment of the 10 patients with *RAS* mutations was 8.5 months (IQR, 4.75–12.00 months). Five patients (50%) exhibited partial response as the optimal response, but 90% of the 10 patients exhibited disease progression during cetuximab-based first-line treatment (Supplementary Table 2). In particular, patient 3 developed *RAS* mutations after 12 months of cetuximab treatment, but disease progression did not occur until the 41st month of follow-up. Only four patients with *RAS* mutations lived until June 30, 2022 (Supplementary Table 2). The median PFS was 8.0 months (IQR, 5.0–12.0 months), and the median OS was 20.0 months (IQR, 8.0–24.0 months).

**Table 3:** Comparisons of the baseline characteristics and clinical outcomes between *RAS* mutant-type and *RAS* wild-type in ctDNA testing for per-protocol populations by Chi-Square test.

	*RAS* mutant-type	*RAS* wild-type	
	(*n* = 10)	(*n* = 98)	
	*n* (%)	*n* (%)	*P*-value
Gender			0.202
Male	8 (80.0)	72 (73.5)	
Female	2 (20.0)	26 (26.5)	
Age (y/o)^a^			0.293
≧65 y/o	6 (60.0)	50 (51.0)	
<65 y/o	4 (40.0)	48 (49.0)	
ECOG^b^ status			9.892
0	2 (20.0)	22 (22.4)	
1	7 (70.0)	76 (77.6)	
2	1 (10.0)	0 (0.0)	
Type of mCRC^c^			1.366
Synchronous	4 (40.0)	58 (59.2)	
Metachronous	6 (60.0)	40 (40.8)	
Primary tumour site			0.882
Right-sided^d^	0 (0.0)	8 (8.2)	
Left-sided^e^	10 (100.0)	90 (91.8)	
Regimen of C/T^f^			1.508
FOLFIRI^g^	10 (100.0)	85 (86.7)	
FOLFOX^h^	0 (0.0)	13 (13.3)	
No.^i^ of metastases			2.014
Only one	7 (70.0)	85 (86.7)	
≧2	3 (30.0)	13 (13.3)	
Best response			0.968
CR	0 (0.0)	0 (0.0)	
PR	5 (50.0)	59 (60.2)	
SD	2 (20.0)	22 (22.4)	
PD	3 (30.0)	17 (17.4)	
ORR			0.391
CR + PR	5 (50.0)	59 (60.2)	
SD + PD	5 (50.0)	39 (39.8)	
DCR			0.963
CR + PR + SD	7 (70.0)	81 (82.6)	
PD	3 (30.0)	17 (17.4)	
Metastatectomy			0.151
Yes	2 (20.0)	15 (15.3)	
No	8 (80.0)	83 (84.7)	
*BRAF* genotyping			0.208
Wild type	10 (100.0)	96 (97.9)	
Mutant type	0 (0.0)	2 (2.1)	

^a^*y/o* year-old.

^b^*ECOG* Eastern Cooperative Oncology Group.

^c^*mCRC* metastatic colorectal cancer.

^d^*Right-sided* including cecum + ascending colon + transverse colon.

^e^*Left-sided* including descending colon + sigmoid colon + rectosigmoid junction + rectum.

^f^C/T: Chemotherapy.

^g^*FOLFIRI* Fluorouracil + leucovorin + irinotecan.

^h^*FOLFOX* Fluorouracil + leucovorin + oxaliplatin.

^i^*No.* Number.

### Primary and secondary endpoints

The percentage of detected *RAS* mutant ctDNA during cetuximab-based first-line treatment was the primary endpoint. Among the 108 per-protocol patients, *RAS* mutant ctDNA was detected in 10 patients with mCRC (9.3%) during cetuximab-base treatment (Table 2). The median duration of cetuximab-based first-line treatment of the 10 patients with mCRC with *RAS* mutations was 8.5 months as one of the secondary endpoints (IQR, 4.75–12.00 months; Supplementary Table 2).

**Fig. 2:**
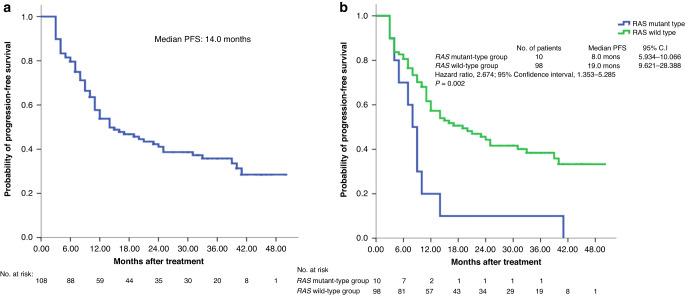
Kaplan–Meier cumulative progression-free survival (PFS) rates. Differences in PFS analysed using log-rank test. **a** Median PFS of 108 patients with metastatic colorectal cancer was 14.0 months. **b** Median PFS of patients with *RAS* mutant ctDNA was significantly inferior to that of patients with *RAS* wild-type ctDNA (8.0 *vs*. 19.0 months, *P* = 0.002).

**Fig. 3:**
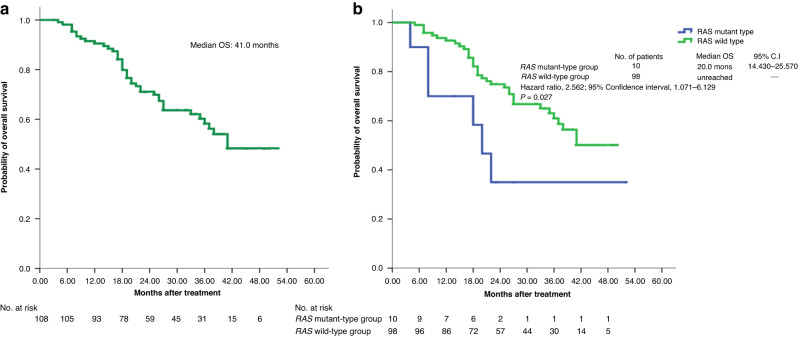
Kaplan–Meier cumulative overall survival (OS) rates. Differences in OS analysed using log-rank test. **a** Median OS of 108 patients with mCRC was 41.0 months. **b** Median OS of patients with *RAS* mutant ctDNA was significantly inferior to that of patients with *RAS* wild-type ctDNA (20.0 months *vs*. unreached, *P* = 0.027).

The results of secondary endpoints showed that the median interval between initial cetuximab-based treatment and *RAS* mutation was 5.0 months (IQR, 2.0–7.5 months; Supplementary Table 2). The median interval between *RAS* mutation and disease progression was 3.0 months (IQR, 2.0–6.5 months; Supplementary Table 2). As shown in Table 2, 48 patients exhibited disease progression during first-line cetuximab-based treatment, and 9 patients with *RAS* mutations exhibited disease progression during first-line cetuximab-based treatment (Supplementary Table 2). The percentage of detected *RAS* mutant ctDNA at the time of progression was 18.8% (9/48). The clinical response rate, as assessed by the investigator per the RECIST criteria, and the metastatic resection rate of patients with mCRC with *RAS* mutations was 59.3% (64/108) and 20.0% (2/10), respectively (Table 2 and Supplementary Table 3). The median PFS and OS of the per-protocol population was 14.0 months and 41.0 months, respectively (Figs. 2a and 3a).

### Comparison of the baseline characteristics, clinical outcomes, PFS and OS between nonmutated (wild) *RAS* populations and acquired mutated *RAS* populations

In Table 3, we observed that the baseline characteristics between the two groups were not significantly different (all *P* > 0.05). The ORR, DCR and metastatectomy were also not significant (*P* = 0.391, 0.963, and 0.151; respectively). Notably, the median PFS was 8.0 and 19.0 months in the *RAS*-mutant and *RAS*-wild groups, respectively (hazard ratio [HR], 2.674; 95% CI, 1.353–5.285; *P* = 0.002; Fig. 2b). The median OS was 20.0 months and unreached in the *RAS*-mutant and *RAS*-wild groups, respectively (HR, 2.562; 95% CI, 1.071–6.129; *P* = 0.027; Fig. 3b). The median PFS and OS were significantly superior in the *RAS*-wild group than in the acquired *RAS*-mutant group.

## Discussion

In the present study, we observed that (1) persistent circulating *RAS* wild-type ctDNA correlated with a greater response duration; (2) emergence in *RAS* mutant ctDNA were related to acquired resistance; (3) the emergence of *RAS* mutant ctDNA enabled early detection and prediction of disease progression; and (4) an upsurge or explosion in *RAS* mutant ctDNA predicted substantial radiological progression. This IIT study also demonstrated that genotyping of *RAS* ctDNA offers clear benefits as a minimally invasive method for indicating tumour heterogeneity.

ctDNA in the bloodstream may be caused by apoptosis, necrosis, or the active secretion of tumour cells [37, 38]. Liquid biopsy, clinically applied to assess existing gene alterations using ctDNA, has been extensively explored for early diagnosis, prediction of recurrence or metastasis, and prognostic value among patients with a variety of cancers [39, 40]. This method is considered transformative and exhibits the benefits of (1) being a noninvasive alternative for identifying solid tumour heterogeneity; (2) enabling assessment of cancer-resistant subclones; and (3) potentially reflecting the molecular dynamics associated with tumour responsiveness and drug resistance [41–44]. The MassARRAY platform has been developed for liquid biopsy applications, and this integrated system may provide benefits in high-throughput detection of multiplex genetic variations [31]. The MassARRAY platform also employs a multigene mutation profiling technique for ctDNA with reasonable sensitivity and specificity to analyse *RAS* mutant ctDNA in patients with mCRC during and after anti-EGFR therapy [45].

Four studies have demonstrated an association between the emergence of circulating *RAS* gene mutations and acquired resistance to anti-EGFR therapies in patients with mCRC [33, 34, 46, 47]. Among patients with *RAS* wild-type mCRC, the detection rate of *RAS* mutant ctDNA was 13–60% when secondary resistance to anti-EGFR treatment was ensured. A study by Misale et al. is highly similar to ours. Both studies were prospective and involved cetuximab-based first-line treatment. The studies differed primarily in the *RAS* ctDNA detection method used, and the enroled patient number in our study was nearly five times that in Misale et al., which reported a detection rate of *RAS* mutant ctDNA of 13%; further, the mutant could be detected as early as 10 months prior to disease progression using radiological documentation [34]. Our data indicated a detection rate of *RAS* mutant ctDNA of 9.3%, and the median detection of disease progression prior to radiological evaluation was 3 months (IQR, 2.0–6.5 months). In another pioneering study by Diaz et al., circulating *KRAS* mutations generally occurred 5–6 months after anti-EGFR therapy [33]. Similarly, the median duration before mutation in our study was 5 months (IQR, 2.0–7.5 months). The relatively small number of enroled patients in these four studies limits their potential value for clinical application. Additionally, the retrospective nature of three of the studies also hinders confidence in the utility of liquid biopsy in monitoring anti-EGFR therapy response. In 2018, Siena et al. mentioned that mutations in *RAS* genes may be a mechanism of secondary resistance in patients with anti-EGFR treatment. Although tumour-tissue biopsy testing has been the standard for evaluating mutational status of *RAS* genes, the plasma testing of cell-free DNA has been shown to be a more sensitive method for detecting clonal evolution [19]. This first prospective analysis in mCRC also showed that serial plasma biopsies are more inclusive than tissue biopsies for evaluating global tumour heterogeneity. To our knowledge, our study, which recruited 120 patients, had the largest patient enrolment among trials of the efficacy of liquid biopsy in patients with mCRC receiving cetuximab as first-line treatment.

Since 2022, three studies regarding anti-EGFR agents induced acquired alternations of ctDNA in patients treated in first-line were published [48–50]. They demonstrated that lower prevalence of acquired genomic alternations by first-line anti-EGFR therapy (6.6–9%), of which is similar to our present findings. Raghav et al. reported that translational relevance to timing and value of ctDNA-guided anti-EGFR rechallenge in patients with mCRC, especially those treated with anti-EGFR therapy upfront [48]. Parseghian et al. supported a model of resistance whereby transcriptomic mechanisms of resistance predominate in the presence of active cytotoxic chemotherapy combined with EGFR inhibitors, with a greater predominance of acquired mitogen-activated protein kinase (MAPK, also called ERK) mutations after single-agent EGFR inhibitors [49]. Vidal et al. demonstrated that ctDNA detected early molecular response and predicted benefit to chemotherapy plus cetuximab. Furthermore, a comprehensive next-generation ctDNA sequencing (NGS) was recommended to integrate information on total disease burden and resistant mutations [50]. In 2015, Siravegna et al. mentioned that the genome of CRC adapts dynamically to pulsatile drug schedules provide rationale for additional lines of therapy for patients who benefit from an initial challenge with anti-EGFR antibodies [51]. The CRICKET trial was designed to prospectively evaluate the activity of a rechallenge strategy with irinotecan plus cetuximab as third-line treatment. Their results demonstrated that a rechallenge strategy with cetuximab and irinotecan may be active in patients with *RAS* and *BRAF* wild-type mCRC with acquired resistance to first-line irinotecan- and cetuximab-based therapy [52]. The ORR and DCR were 21% and 54%, respectively.

After a median follow-up of 26.5 months, the subgroup with *RAS* mutant ctDNA exhibited significantly lower median PFS and median OS than did the subgroup with *RAS* wild-type ctDNA. Compared with those of patients with FIRE-3 [53] and CALGB/SWOG 80405 [54], the median PFS (8 *vs*. 10 *vs*. 10.5 months) and median OS (20.0 *vs*. 28.7 *vs*. 30.0 months) of the subgroup with *RAS* mutant ctDNA were inferior. In addition to demonstrating that *RAS* wild-type ctDNA levels are a potential biomarker for continued response to cetuximab-based therapy, this study also demonstrated that highly and rapidly increasing *RAS* mutant ctDNA levels are a potential biomarker of poor prognosis because these increases were followed by imminent clinical deterioration and the spread of metastases. Our study has the following limitations: (1) 12 patients (10.0%) were excluded because of incompletion in the study, and none of the patients received more than 6-cycle treatment, so no any post-treatment imaging data were evaluated; (2) the analysis of data only comes from per-protocol populations rather than from intention-to-treat populations; (3) the physicians were not blinded for response assessment, and quality of life questionnaires were not used in this study; (4) the follow-up time was relatively short, and an evaluation of the long-term efficacy of treatment, especially in terms of OS, was not available.

The results of this prospective study demonstrated that the MassARRAY platform is a system for tumour genotyping to assess the emergence of *RAS* mutations to evaluate resistance to anti-EGFR treatment. Moreover, in this proof-of-concept prospective study of liquid biopsy monitoring, we demonstrated that continued circulating *RAS* wild-type ctDNA status is a valuable biomarker for prolonged tumour response to anti-EGFR therapy, and that *RAS* mutation emergence events were used to predict resistance to anti-EGFR therapy and an imminent radiological progression in patients with mCRC.

## Supplementary information

Supplementary figure 1 legend

STROBE statement

Supplementary table 1

Supplementary table 2

Supplementary figure 1

## Data Availability

The data and materials analysed in the current study are available from the corresponding authors on reasonable requests.
