# Supplementary material for: The emergence of RAS mutations in patients with RAS wild-type mCRC receiving cetuximab as first-line treatment: a noninterventional, uncontrolled multicenter study
Source: Br J Cancer. 2023 Jul 24;129(6):947–55. doi: 10.1038/s41416-023-02366-z (PMC10491612; doi:10.1038/s41416-023-02366-z)
Supplement: Supplementary file 1 — Supplementary figure 1 legend [file 41416_2023_2366_MOESM1_ESM.docx]

**SUPPLEMENTARY FIGURE LEGENDS**

**Supplementary Fig. 1. The brief overview of the protocol diagram.**
