# Supplementary material for: The emergence of RAS mutations in patients with RAS wild-type mCRC receiving cetuximab as first-line treatment: a noninterventional, uncontrolled multicenter study
Source: Br J Cancer. 2023 Jul 24;129(6):947–55. doi: 10.1038/s41416-023-02366-z (PMC10491612; doi:10.1038/s41416-023-02366-z)
Supplement: Supplementary file 3 — Supplementary table 1 [file 41416_2023_2366_MOESM3_ESM.docx]

**Supplementary table 1.** Baseline characteristics of 10 metastatic colorectal cancer (mCRC) patients with *RAS* gene mutations during the period of cetuximab-based as first-line treatment

| Number | Gender | Age (years old) | Mutation point (*KRAS*) | Primary site | Sidedness | Type of mCRC^1^ | Chemotherapy regimen |
| --- | --- | --- | --- | --- | --- | --- | --- |
| 1 | male | 63 | G13D | rectum | left-sided | metachronous | FOLFIRI |
| 2 | male | 51 | Q61K | sigmoid | left-sided | synchronous | FOLFIRI |
| 3 | male | 69 | G12D | rectum | left-sided | metachronous | FOLFIRI |
| 4 | female | 70 | K117N | rectum | left-sided | synchronous | FOLFIRI |
| 5 | male | 72 | G13D | rectum | left-sided | metachronous | FOLFIRI |
| 6 | female | 76 | A59T | sigmoid | left-sided | metachronous | FOLFIRI |
| 7 | male | 62 | Q61H | rectum | left-sided | metachronous | FOLFIRI |
| 8 | male | 69 | G12D | rectum | left-sided | synchronous | FOLFIRI |
| 9 | male | 61 | G12D | rectum | left-sided | synchronous | FOLFIRI |
| 10 | male | 67 | G12D | sigmoid | left-sided | metachronous | FOLFIRI |

^1^mCRC: metastatic colorectal cancer;
