# Supplementary material for: The emergence of RAS mutations in patients with RAS wild-type mCRC receiving cetuximab as first-line treatment: a noninterventional, uncontrolled multicenter study
Source: Br J Cancer. 2023 Jul 24;129(6):947–55. doi: 10.1038/s41416-023-02366-z (PMC10491612; doi:10.1038/s41416-023-02366-z)
Supplement: Supplementary file 4 — Supplementary table 2 [file 41416_2023_2366_MOESM4_ESM.docx]

**Supplementary table 2.** Clinical outcomes of 10 metastatic colorectal cancer (mCRC) patients with *RAS* mutations

| No.^1^ | Duration of Cetuximab treatment (months) | Best response | Metastatectomy | Progression during 1st-line treatment | PFS^5^ (months) | Survival^6^ | OS^7^ (months) | Interval between initial therapy to *RAS* mutation (months) | Interval between *RAS* mutation to progressive disease (months) |
| --- | --- | --- | --- | --- | --- | --- | --- | --- | --- |
| 1 | 9 ms | SD^2^ | no | yes | 9 ms | yes | 23 ms | 7 ms | 2 ms |
| 2 | 4 ms | PD^3^ | no | yes | 3 ms | no | 4 ms | 2 ms | 2 ms |
| 3 | 12 ms | PR^4^ | yes | no | 41 ms* | yes | 52 ms | 15 ms | 26 ms |
| 4 | 5 ms | PD^3^ | no | yes | 5 ms | no | 8 ms | 2 ms | 3 ms |
| 5 | 12 ms | PR^4^ | no | yes | 12 ms | yes | 27 ms | 9 ms | 3 ms |
| 6 | 8 ms | SD^2^ | no | yes | 8 ms | yes | 14 ms | 6 ms | 2 ms |
| 7 | 4 ms | PD^3^ | no | yes | 4 ms | no | 8 ms | 2 ms | 2 ms |
| 8 | 14 ms | PR^4^ | no | yes | 14 ms | no | 20 ms | 6 ms | 8 ms |
| 9 | 10 ms | PR^4^ | no | yes | 10 ms | no | 18 ms | 4 ms | 6 ms |
| 10 | 7 ms | PR^4^ | yes | yes | 7 ms | no | 22 ms | 4 ms | 3 ms |

^1^No: Number; ^2^SD: Stable disease; ^3^PD: Progressive disease; ^4^PR: Partial response; ^5^PFS: Progression-free survival within the period of follow-up till May-2022; ^6^Survival: alive till May-2022; ^7^OS: Overall survival.

*This patient was no progressive disease within the period of cetuximab treatment but within the follow-up time.
