# Supplementary figures and images for: The emergence of RAS mutations in patients with RAS wild-type mCRC receiving cetuximab as first-line treatment: a noninterventional, uncontrolled multicenter study
Source: Br J Cancer. 2023 Jul 24;129(6):947–55. doi: 10.1038/s41416-023-02366-z (PMC10491612; doi:10.1038/s41416-023-02366-z)

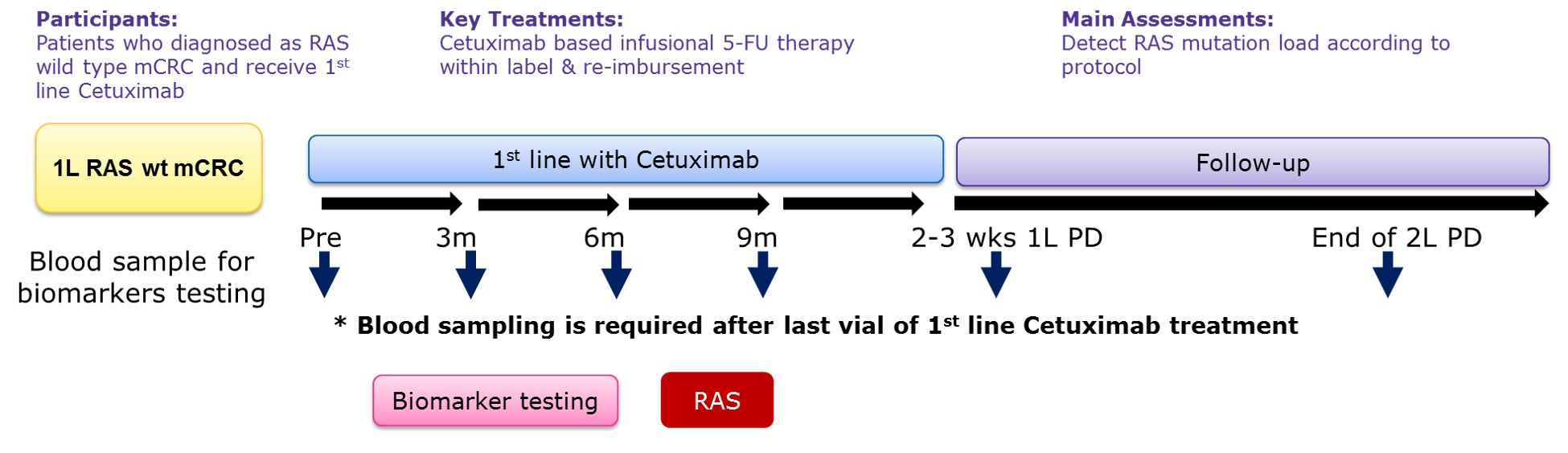

Supplement: Supplementary file 5 — Supplementary figure 1 [file 41416_2023_2366_MOESM5_ESM.tif]
